# Supplementary material for: Long COVID: A Systematic Review of Preventive Strategies
Source: Infect Dis Rep. 2025 May 21;17(3):56. doi: 10.3390/idr17030056 (PMC12101273; doi:10.3390/idr17030056)
Supplement: Supplementary file 1 [file idr-17-00056-s001.zip › idr-3575952-supplementary.pdf]

infectious disease reports

MDPI

Table S1. Impact of COVID-19 vaccination on prevention of long COVID: studies that defined long COVID as symptoms lasting more than 28 days.

Vaccine: Protective	
**Study Design**	**Study Details**
**Prospective**	**Azzolini et al. [1]:** Italy, March 2020-Apr 2022 (wild type, A, Δ, O); non-hospitalized HCW (n=739), F (75%); LC, Sx ≥ 28d; vaccinated with one dose of BNT162b2, aOR: 0.86 (95% CI: 0.21-3.49); two doses, aOR: 0.25 (0.07-0.87); three doses, aOR: 0.16 (0.03-0.84) vs. unvaccinated.
	**Di Fusco et al. [2]:** USA CVS Health Test Sites, nationwide survey-based PRO study; Jan-April 2022 (O); outpatients (n=328), F (73.8%); LC, 3 or more Sx ≥ 28d; vaccine type (BNT162b2); boosted, aOR: 0.22 ($P < 0.001$), primary series, aOR: 0.74 (P=0.332) vs. unvaccinated.
**Retrospective**	**Xie et al. [3]:** USA VHA database, March 2020-Jan 2022 (wild type, A, Δ, O); COVID-19 positive (n=441,583), non-infected (n=4,748,504), F (10%); LC defined based on ICD-10 codes between 30d and 1 year after COVID-19; vaccine type (BNT162b2, mRNA-1273, or Ad26.COV2.S); 78.9% reduction in LC attributable to vaccines (95% CI: 69.5-74.4) and 28.1% (25.6-30.5) attributable to era-related effects (changes in virus and other temporal effects).
	**Lundberg-Morris et al. [4]:** Sweden, nationwide linked multiregister project (SCIFI-PEARL), Dec 2020-Feb 2022 (wild type, A, Δ, O); participants (n = 589,722, covering ~40% of Sweden's population), vaccinated (50.8%), F (53%); LC, Sx ≥ 28d; vaccine type (BNT162b2, mRNA-1273, Ad26.COV2.S, ChAdOx1, or NVX-CoV2373); any n of vaccines before infection, aHR: 0.42 (95% CI: 0.38-0.46); one dose, aHR: 0.79 (0.68-0.91), two doses, aHR: 0.41 (0.37-0.45), three doses, 0.27 (0.23-0.32) vs. no vaccines.
	**Malden et al.[5]:** USA, 1:1 matched cohort, March 2021-Feb 2022 (A, Δ, O); participants (n=323,062), F (54.5%); LC, 13 categories of Sx from 30 d to 6 mo after COVID-19; vaccine type (BNT162b2 or mRNA-1273); vaccinated with reduced symptoms: non-specific COVID-19-related disorders, RR: 0.53 (95% CI: 0.51–0.56); skin and subcutaenous tissue disorders, RR: 0.69 (0.66-0.72); blood and hematologic disorders, RR: 0.79 (0.71-0.89); circulatory system disorders, RR: 0.88 (0.83-0.94); sensory disorders, RR: 0.90 (0.86-0.95); all disorders except for mental health disorders, RR: 1.06 (1.02-1.10).
	**Al-Aly et al. [6]**: USA VHA database, Jan-Oct 2021 (A, Δ); patients (n=147,414), F (8-10%); LC, Sx ≥ 30d; vaccine types (BNT162b2, mRNA-1273, or Ad26.COV2.S); BTI (fully vaccinated[a]) vs. unvaccinated, HR: 0.85 (95% CI: 0.58-0.89).
	**Tannous et al. [7]** USA Houston Methodist CURATOR, March 2020-Nov 2021 (wild type, A, Δ); patients (n=53,239), F (54.9%); LC, Sx ≥ 28d; vaccine types (BNT162b2, mRNA-1273, or Ad26.COV2.S); fully vaccinated[a] vs. unvaccinated, aOR: 0.58 (95% CI: 0.52-0.66); mAb vs. no mAb after COVID-19 infection, aOR: 0.77 (95% CI: 0.69-0.86).
	**Brannock et al. [8]:** USA RECOVER initiative, data collected from EHR through National COVID Cohort Collaborative, Aug 2021-Jan 2022 (Δ, O); clinic-based cohort patients (n= 47,404), F (65%); LC, Sx ≥ 45d; fully vaccinated[a] vs. unvaccinated, proportional HR: 0.67 (95% CI: 0.56-0.79).
	**Ayoubkhani et al. [9]:** UK, large national survey CIS, Apr 2020-Nov 2021 (wild type, A, Δ); 1:1 matched (n=3090 in each arm), F (57.7%); LC, Sx ≥ 28d; vaccine types (BNT162b2, mRNA-1273, or AZD1222); vaccinated with 2 doses vs. unvaccinated, aOR: 0.59 (95% CI: 0.50-0.69).
	**Sigler et al. [10]:** USA UCSD data collected from EMR; June 2020-April 2022 (wild type, A, Δ, O); LC, Sx ≥ 6 wks; SOTR (n=208), F (36%); vaccine type (BNT162b2, mRNA-1273, Ad26.COV2.S); at least one dose of vaccination prior to transplant, aOR: 0.39 (95% CI: 0.16-0.95).
**Vaccine: Not Protective**	
**Retrospective**	**Taquet et al. [11]:** EHR (TriNetX network, mostly patients in USA), Jan-Aug 2021 (A, Δ); F (59.4%); LC, Sx ≥ 28d within 6 mo after COVID-19; matched cohorts (9479 vaccinated with at least one dose of BNT162b2 (65.1%), mRNA-1273 (9.0%), or Ad26.COV2.S (1.6%) 2 wks before COVID-19 vs. 9,479 unvaccinated), HR: 1.01 (95% CI: 0.96-1.05)

fully vaccinated[a], two doses of BNT162b2 or mRNA-1273 or a single dose of Ad26.COV2.S 2 weeks prior to COVID-19.

Abbreviations; USA, United States of America; UK, United Kingdom; HCW, health care worker; n, number; F, female; LC, long COVID; PRO, patient-reported outcome; VHA, Veterans Health Administration; Univ, university; CIS, COVID-19 Infection Survey; EMR, electronic medical record; CURATOR, COVID-19 Surveillance and

Outcomes Registry; UCSD, University of California, San Diego; Jan, January; Feb, February; Apr, April; Aug, August; Oct, October; Nov, November; Dec, December; d, days; wks, weeks; mo, months; EHR, electronic health records; SOTRs, solid organ transplant recipients; A, Alpha; Δ, Delta; O, Omicron; ICD-10, International Classification of Diseases 10th revision;; Sx, symptoms; BTI, breakthrough infection; mAb, monoclonal antibody; aOR, adjusted odds ratio; CI, confidence interval; aHR, adjusted hazard ratio; RR, risk ratio.

Table S2. Impact of antivirals on prevention of long COVID: studies that defined long COVID as symptoms lasting more than 28 days.

Antivirals: Protective	
**Study Design**	**Study Details**
**Retrospective**	**Fung et al. [12]:** USA, medicare database for enrollees aged ≥ 65 years, Jan-Sep 2022 (O); COVID-19 patients (n=3,975,690), F (60.8%), nirmatrelvir (19.5%), molnupiravir (2.6%); LC, Sx 4-12wks; nirmatrelvir, aHR: 0.87 (95% CI: 0.86-0.88); molnupiravir, aHR: 0.92 (95% CI: 0.90-0.94).
	**Xie et al. [13]:** USA VHA database, Jan 2022-Jan 2023 (O); COVID-19 outpatients (n=281,793), F (12.2%); LC, Sx 31-180d; treated with nirmatrelvir within 5d of COVID-19, aRR: 0.74 (95% CI: 0.72-0.77).
	**Xie et al. [14]:** USA VHA database, Jan-Dec 2022 (O); COVID-19 outpatients (n=229,286), molnupiravir (5%), F (8.4%); LC, Sx 31-180d; treated with molnupiravir within 5d of COVID-19, aRR: 0.86 (95% CI: 0.83-0.89).
**Antivirals: Not Protective**	
**Retrospective**	**Loannou et al. [15]:** USA VHA database, 1:1 matched cohort, Jan-July 2022 (O); LC, Sx 31-180d; outpatients, each group (n=9593), F (14%); no differences between nirmatrelvir/ritonavir treated within 5d of COVID-19 and untreated groups, but lower thromboembolism in nirmatrelvir/ritonavir-treated group, sHR: 0.65 (95% CI: 0.44-0.97).

Abbreviations; USA, United States of America; VHA, Veterans Health Administration; ; Jan, January; Sep, September; Dec, December; n, number; F, female; A, Alpha; Δ, Delta; O, Omicron; LC, long COVID; Sx, symptoms; d, days; wks, weeks; mo, months; aHR, adjusted hazard ratio; aRR, adjusted risk ratio; sHR, subhazard ratio; CI, confidence interval.

References

1. Azzolini E, Levi R, Sarti R, Pozzi C, Mollura M, Mantovani A, Rescigno M. Association Between BNT162b2 Vaccination and Long COVID After Infections Not Requiring Hospitalization in Health Care Workers. Jama 2022; 328(7): 676-8.
2. Di Fusco M, Sun X, Moran MM, Coetzer H, Zamparo JM, Alvarez MB, Puzniak L, Tabak YP, Cappelleri JC. Impact of COVID-19 and effects of booster vaccination with BNT162b2 on six-month long COVID symptoms, quality of life, work productivity and activity impairment during Omicron. J Patient Rep Outcomes 2023; 7(1): 77.
3. Xie Y, Choi T, Al-Aly Z. Postacute Sequelae of SARS-CoV-2 Infection in the Pre-Delta, Delta, and Omicron Eras. N Engl J Med 2024; 391(6): 515-25.
4. Lundberg-Morris L, Leach S, Xu Y, Martikainen J, Santosa A, Gisslen M, Li H, Nyberg F, Bygdell M. Covid-19 vaccine effectiveness against post-covid-19 condition among 589 722 individuals in Sweden: population based cohort study. BMJ 2023; 383: e076990.
5. Malden DE, Liu IA, Qian L, Sy LS, Lewin BJ, Asamura DT, Ryan DS, Bezi C, Williams JTB, Kaiser R, et al. Post-COVID conditions following COVID-19 vaccination: a retrospective matched cohort study of patients with SARS-CoV-2 infection. Nat Commun 2024; 15(1): 4101.
6. Al-Aly Z, Bowe B, Xie Y. Long COVID after breakthrough SARS-CoV-2 infection. Nat Med 2022; 28(7): 1461-7.
7. Tannous J, Pan AP, Potter T, Bako AT, Dlouhy K, Drews A, Sostman HD, Vahidy FS. Real-world effectiveness of COVID-19 vaccines and anti-SARS-CoV-2 monoclonal antibodies against postacute sequelae of SARS-CoV-2: analysis of a COVID-19 observational registry for a diverse US metropolitan population. BMJ Open 2023; 13(4): e067611.
8. Brannock MD, Chew RF, Preiss AJ, Hadley EC, Redfield S, McMurry JA, Leese PJ, Girvin AT, Crosskey M, Zhou AG, et al. Long COVID risk and pre-COVID vaccination in an EHR-based cohort study from the RECOVER program. Nat Commun 2023; 14(1): 2914.

9. Ayoubkhani D, Bosworth ML, King S, Pouwels KB, Glickman M, Nafilyan V, Zaccardi F, Khunti K, Alwan NA, Walker AS. Risk of Long COVID in People Infected With Severe Acute Respiratory Syndrome Coronavirus 2 After 2 Doses of a Coronavirus Disease 2019 Vaccine: Community-Based, Matched Cohort Study. Open Forum Infect Dis 2022; 9(9): ofac464.
10. Sigler R, Covarrubias K, Chen B, Rubarth RB, Torosian K, Sanchez CR, Bharti A, DeGruttola V, Aslam S. Post-acute sequelae of COVID-19 in solid organ transplant recipients. Transpl Infect Dis 2023; 25(6): e14167.
11. Taquet M, Dercon Q, Harrison PJ. Six-month sequelae of post-vaccination SARS-CoV-2 infection: A retrospective cohort study of 10,024 breakthrough infections. Brain Behav Immun 2022; 103: 154-62.
12. Fung KW, Baye F, Baik SH, McDonald CJ. Nirmatrelvir and Molnupiravir and Post-COVID-19 Condition in Older Patients. JAMA Intern Med 2023; 183(12): 1404-6.
13. Xie Y, Choi T, Al-Aly Z. Association of Treatment With Nirmatrelvir and the Risk of Post-COVID-19 Condition. JAMA Intern Med 2023; 183(6): 554-64.
14. Xie Y, Choi T, Al-Aly Z. Molnupiravir and risk of post-acute sequelae of covid-19: cohort study. BMJ 2023; 381: e074572.
15. Ioannou GN, Berry K, Rajeevan N, Li Y, Mutalik P, Yan L, Bui D, Cunningham F, Hynes DM, Rowneki M, et al. Effectiveness of Nirmatrelvir-Ritonavir Against the Development of Post-COVID-19 Conditions Among U.S. Veterans : A Target Trial Emulation. Ann Intern Med 2023; 176(11): 1486-97.
